# Atraumatic Acromioclavicular Joint Dislocation: A Case Report Treated with Excision of the Distal Clavicle Margin and Button Slide System with Allograft Tendon Reinforcement at Coracoclavicular and Acromioclavicular Joint

**DOI:** 10.3390/jpm12122043

**Published:** 2022-12-10

**Authors:** Alejandro León, Sergio Chavez, Belén Garcia-Medrano, Rubén García-Fraile, Pablo Beltrán de Heredia, Jesús Palencia, Alberto Caballero-García, Alfredo Córdova, David Noriega Gonzalez

**Affiliations:** 1 Orthopaedics and Traumatology Surgery, Hospital Clínico Universitario de Valladolid (HCUV), 47005 Valladolid, Spain; 2 Orthopaedics and Traumatology Surgery, Hospital Universitario Rio Hortega, 47012 Valladolid, Spain; 3 Department of Anatomy and Radiology, Health Sciences Faculty, GIR: “Physical Exercise and Aging”, University of Valladolid, Campus Universitario “Los Pajaritos”, 42004 Soria, Spain; 4 Department of Biochemistry, Molecular Biology and Physiology, Health Sciences Faculty, GIR: “Physical Exercise and Aging”, University of Valladolid, Campus Universitario “Los Pajaritos”, 42004 Soria, Spain

**Keywords:** acromioclavicular, allograft, augmented repair, atraumatic

## Abstract

Although acromioclavicular joint (ACJ) dislocation is a common injury following trauma involving the shoulder, it is rare in the absence of trauma. In this manuscript, we describe a case of ACJ in a 15-year-old girl who presented a painful dislocation with spontaneous shortening of the right acromioclavicular joint that forced her to temporarily abandon her sports career. After failure of conservative physiotherapy treatment, surgical intervention was proposed by performing an arthroscopic-assisted button slide combined with augmented hamstring allograft reconstruction. After the intervention and the subsequent recovery period, the athlete was able to return to her semi-professional training. The follow-up of the patient is 5.5 years post-surgery. The result obtained could help in planning the treatment of future cases.

## 1. Introduction

Dislocation of the acromioclavicular joint (ACJ) accounts for about 9% of shoulder injuries in the general population and increases to 40% among athletes participating in highly competitive impact sports [1].

The acromioclavicular joint is a flat joint. Its capsule is reinforced by the superior and inferior acromioclavicular ligaments, of which the first is the strongest structure. These ligaments are primarily responsible for restricting (stabilizing) the posterior displacement of the outer end of the clavicle [2]. The stability of the ACJ joint therefore depends primarily on the coracoclavicular and acromioclavicular ligaments. This ACJ dislocation involves an injury to the acromioclavicular ligament with or without rupture of the coracoclavicular ligament [3]. The ACJ is involved in flexion, extension, and sliding where the space between the acromion and clavicle increases or decreases. However, the main movement that takes place in the joint is rotation in the long axis of the clavicle [4].

According to Murena et al. [5], patients with severe instability are at risk of developing anatomical changes in scapular alignment and kinematics that may lead to impaired rotator cuff function. Fukuda et al. [2] have suggested that obliquity of the articular surface may predispose one to traumatic disruption. The mechanism of injury has been suggested to be forced depression of the acromion due to a fall onto the point of the shoulder [6].

Janecki [7] and Richards et al. [8] have pointed to hyperlaxity as the main cause of these situations. Janecki was the first to describe a patient with voluntary dislocation of the ACJ; although not confirmed, the patient had features compatible with a connective tissue disorder. Later, Richard et al. [8] described another asymptomatic dislocation in a patient previously diagnosed with cerebral palsy, causing hemiparesis and associated hyperlaxity.

After an injury, the patient has pain and swelling in the shoulder region and increased tenderness over the ACJ. If no step can be palpated on the superior aspect of the acromioclavicular joint, it is likely that the patient has only suffered a sprain.

Atraumatic ACJ is a very rare condition, with only five cases described in the literature previously. These five cases were all voluntary, i.e., not atraumatic [7,8,9,10,11].

The following is a case of an adolescent athlete with atraumatic AC dislocations of the right shoulder treated with excision of the distal clavicle margin and button slide system adding allograft tendon plasty to cocacoclavicular and acromioclavicular joint.

## 2. Case Report

This case is about a young female, 15 years old, who comes to the clinic with a history of pain in the right shoulder with clicking noises during movements that exceed the level of the head, worsening with sports practice. The patient does not refer to any traumatic event, nor does she relate it to any specific moment.

The patient had been practicing rhythmic gymnastics for 5 years, a sport she practiced at a high competition level. She first came to the clinic with the following symptoms: pain in the right shoulder, predominantly in the anterosuperior aspect, with clicking noises, sensation of lack of strength in the shoulder during repeated flexion and separation, with abnormal movement and scapulothoracic dyskinesia in the region of the right acromioclavicular joint. 

Right acromioclavicular instability was diagnosed from the outset, and conservative treatment based on physiotherapy was established. However, after 6 months and due to the recurrence of symptoms, the patient abandoned the competition.

During her evolution, the patient have presented pain in the right shoulder, predominantly in the anterosuperior aspect, with a clicking sensation and a feeling of lack of strength during repeated flexion and separation. At the beginning she had no pain, but over the following months, she presented limiting pain of mechanical characteristics.

On physical examination, there was evident anteroposterior acromioclavicular instability on active flexion of the arm. A right scapulothoracic dyskinesia was also very evident when flexing or separating the right arm. The patient had full range of motion of the shoulder.

On clinical examination of the shoulder, posterior stress tests were performed using the Sulcus, Jerk and Drawer tests, which were negative. There was no limitation of range of motion (ROM) of the shoulder. A significant scapulothoracic dyskinesia (SD) was observed, especially during flexion and abduction movements. 

Likewise, following the protocol published by Fernandez et al. [12], the relevant exploratory tests of the shoulder were performed. The Yocum, Hawkins and Empty maneuvers were positive, and the Beighton score was also positive.

On palpation, at rest, a slight prominence of the AC joint was noted, with increased tenderness around it. A slight increase in posterior translation was noted, but with a clear end point. Anterior flexion resulted in painful antero-posterior dislocation with spontaneous reduction at rest. In addition, the patient could perform voluntary AC dislocation by means of a mechanism of ante-retropulsion scapular movement.

**Figure 1:**
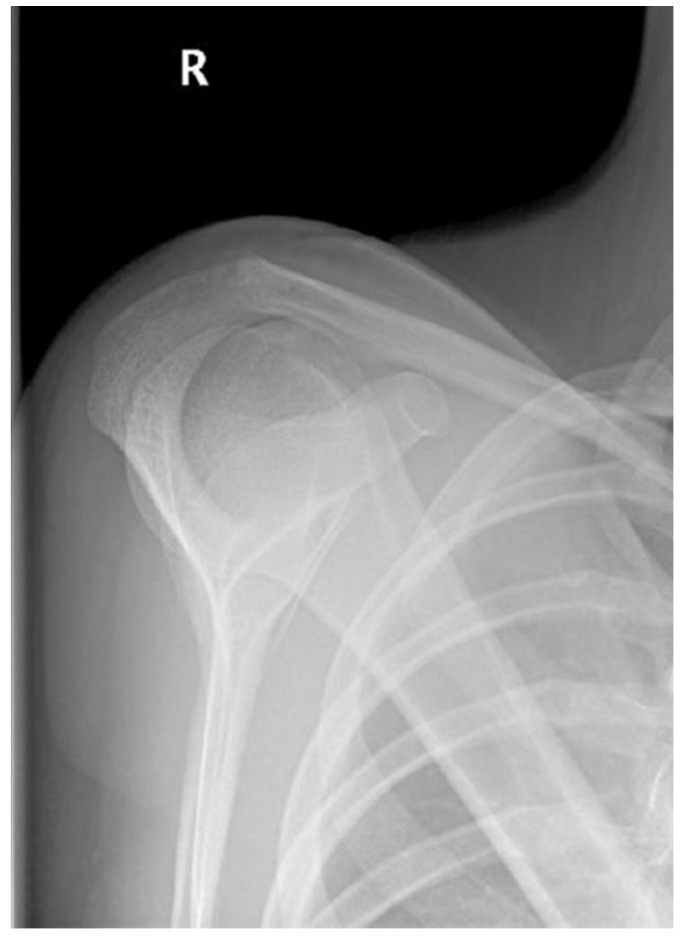
Clavicle shortening in Alexander projection, by overlap.

As for the radiographic examination, nothing remarkable was observed in the conventional anteroposterior (AP) projection, although in the Alexander projection, an asymmetry between both shoulders was observed, with shortening (Figure 1). 

**Figure 2:**
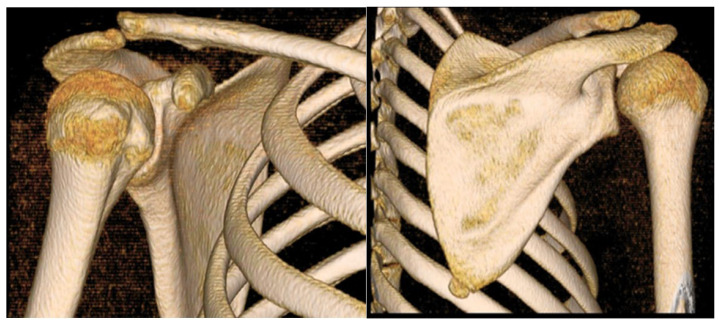
Preoperative 3D (vision) reconstruction CT scan, with posterior and upper clavicle subluxation.

**Figure 3:**
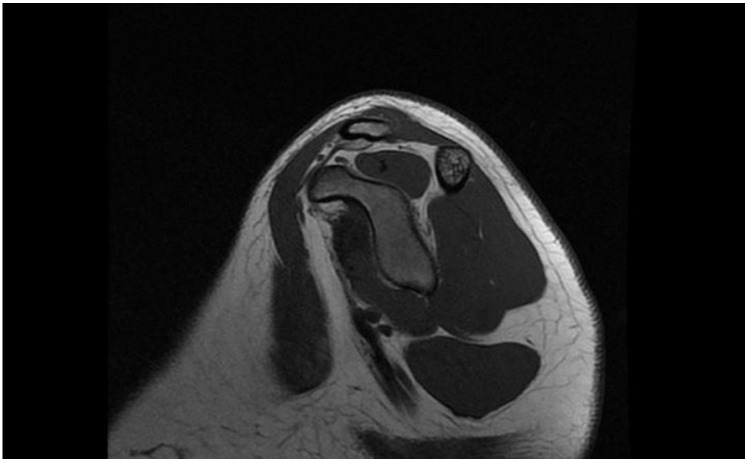
Trapezoidal ligament integrity in preoperative magnetic resonance imaging (MRI).

The ultrasound study showed dynamic instability of the acromioclavicular (AC), without tendon lesions or other pathological findings of soft parts. Computed axial tomography (CT) showed a superior subluxation of 5 mm (Figure 2) and magnetic resonance imaging (MRI) showed the integrity of the conoid and trapezoid ligaments (Figure 3).

Prior to surgery, and at the patient’s request, conservative treatment was followed for 9 months, consisting of neuromuscular conditioning and deltoid, serratus and pectoral strengthening program, trying to correct scapulothoracic dyskinesia. After the failure of the conservative treatment, surgical intervention was proposed as the symptoms did not disappear.

The aim of the surgical approach was to provide stability of the acromioclavicular joint in the vertical and horizontal planes, and to reinforce the coracoclavicular ligaments and the acromioclavicular capsulo-ligamentous complex. Therefore, the intervention should provide adequate primary stability to avoid prolonged joint immobilization as well as provide secondary stability. Thus, it was decided to provide biological support with tendon grafting. 

Preservation of the articular capsuloligamentary structures allows preservation of joint function in the short and long term.

## 3. Treatment

In the “beach chair” position, an arthroscopic time was first performed, which showed no articular or labral lesions and exposed the coracoid process. A 5 cm long incision was then made following the clavicle from the ACJ. During the surgical process, careful hemostasis was performed for adequate dissection of the deltoid and soft tissues.

Subsequently, coracoclavicular and acromioclavicular joint plasty was planned. The procedure followed was as follows.

The distal margin of the clavicle was removed and then, with arthroscopic and radiographic assistance, a drill was made in the distal clavicle (about 30 mm from its end) and in the coracoid process after a correct shortening of the acromioclavicular. Drilling was performed with a 4.5 mm drill bit, cannulated, in both bones. This drilling was performed over a 2.4 mm guide pin. 

A suture button device (Ziptight, Zimmer-Biomet, Warsaw, IN, USA) was passed through the hole made, and by alternating traction of the double self-locking loop sutures, shortening of the ACJ and correct button placement was achieved. 

**Figure 4:**
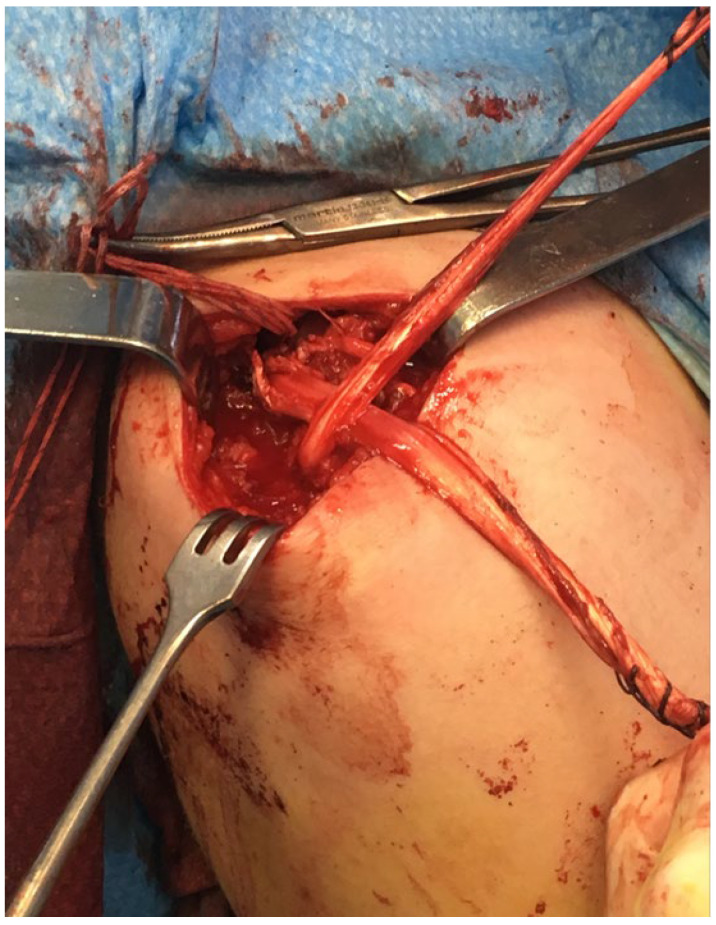
Intraoperative image of the tendon graft through the acromion and crossing the clavicle.

The semitendinosus allograft was then prepared to fit the hole (4.5 mm). For this purpose, both ends of the allograft were sutured with Krakow-type sutures. The distal clavicle and acromion were drilled using the same broaching system used for the coracoclavicular (about 15 mm from both bony ends of the acromioclavicular), and then the intraosseous plasty was passed, tensioned and sutured with high-strength thread between both tendon ends of the allograft on the anterosuperior aspect of the shoulder (Figure 4).

The deltoid fascia was properly repaired after generous saline irrigation of the wound. Wound skin closure was performed with 3/0 resorbable suture. The limb was placed in a Gilchrist sling for 4 weeks, but assisted flexion of the arm was encouraged from the outset. 

**Figure 5:**
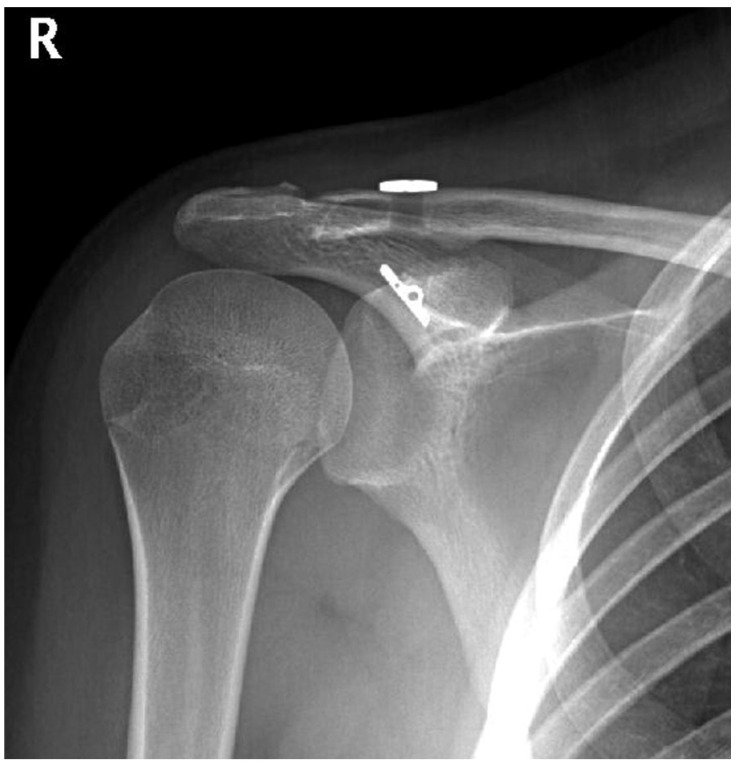
Postoperative radiographic control, with hyper shortening of the acromioclavicular joint.

After the surgical process, at the first visit, after 2 weeks of ACJ, a radiological examination was performed and showed a shortening with an overcorrection of 5 mm (Figure 5).

Six weeks after surgery, the patient had no pain, and showed a range of motion of 150° for abduction and flexion, and no limitation for rotation or extension; therefore, she was referred to the rehabilitation service for the correct physiotherapeutic treatment. It was remarkable that scapulothoracic dyskinesia disappeared at 6 weeks of follow-up.

At 2 months after surgery, full range of motion was achieved, without pain, with a stable ACJ and a displacement of 0 mm on imaging control.

**Figure 6:**
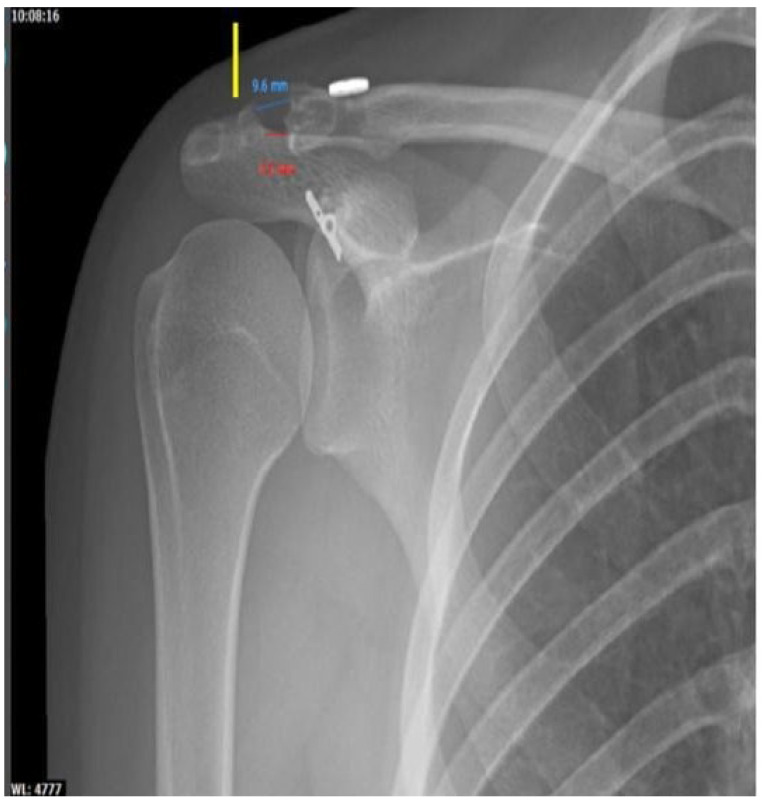
5.5 years post-surgery radiographic control. Correct acromioclavicular alignment, with increased acromioclavicular space. Yellow arrow: vertical bone tunnel of the tendon plasty.

Six months after surgery, the patient was able to return to her previous sports practice. Currently, after more than 5 years of follow-up, she remains an amateur dancer with an American Shoulder and Elbow Surgeon score of 87 and a Constant score of 86. From the radiological point of view, it can be observed that a perfect acromioclavicular shortening is maintained, without having increased a minimal osteolysis of the external clavicular third, a fact that was first observed two years after surgery (Figure 6).

## 4. Discussion

The choice of the treatment performed for the resolution of this case was not accidental, as we tried to explain the biomechanical reasons that led the patient to be treated in this way, and without the existence of previous published case reports with the same diagnosis as ours and which followed the same treatment as ours.

Our case is the only one with this pathology treated with this surgical technique (described below), with more than five years of follow-up, a period of time that would be sufficient to develop potential complications that can arise from this surgical technique. None of the other five previously published cases [13,14,15,16,17] had as successful clinical and functional outcomes as have been achieved in our case, with complete reincorporation of daily activities and without relapses or complications.

An understanding of glenohumeral anatomy and biomechanics plays a key role in detecting dynamic or static structures that are affected so that appropriate treatment can be achieved [18].

The constant technological progress has been a fundamental part of continuing the development of new techniques in open or arthroscopic surgery, as well as implants. This has achieved satisfactory results without compromising the function of other adjacent structures.

In the case we present, a relevant fact is the success of the technique used, because after the continuous follow-up of the patient, in the last one performed (at 5 years), we observed a perfect acromioclavicular congruence (disappearance of the initial postoperative hypercorrection). There was no osteolysis around the osteosynthesis material and only minimal osteolysis of the outer third of the clavicle was observed with a joint space of 6 mm in the most cranial area of the same and 9 mm in the upper area of the same.

All this is relevant, since we must remember that, in the case presented, the etiology was not clear, although it is true that the conoid and trapezoidal ligaments maintained their integrity. 

Of the five previous cases, three were treated orthopedically without success. The two cases treated surgically also did not have a satisfactory result. In the first one, the coracoid transfer osteotomy was complemented with a resection of the external third of the clavicle. In our opinion, this surgical technique does not provide stability in the horizontal plane. Biomechanically, the clavicular stabilization by means of a single screw provides a primary fixation evidently inferior to the endobutton-type system, and it does not act directly in stabilizing the acromioclavicular joint in the horizontal plane [13,14,15,16,17]. 

In Sahara et al.’s research [11], a 19-year-old woman with an atraumatic posterior dislocation was treated with excision of the distal clavicle combined with transfer of the coracoid tip and joint tendon to the clavicle. 

Barchick et al. [10] reported another patient with bilateral dislocation. In this case, the patient was treated with coracoclavicular ligament repair, augmented by arthroscopy for the right shoulder, and conservative treatment for the left. Interestingly, they obtained similar results in both extremities. However, they did observe that intense pain was maintained in those activities that required passing the arms over 90º of flexion or separation.

When facing the surgery in the case we present, we did not perform it bilaterally, but exclusively unilaterally, different to the treatment applied by other authors [7,8,9,11]. In the case we studied, a double fixation was performed, first through primary vertical coracoclavicular stabilization with endobutton system, and later stabilization with intraosseous biological support of the acromioclavicular and coracoclavicular tendon, which allows stabilization in the vertical and horizontal plane.

One of the objectives of arthroscopy is to check for intra-articular lesions. In our case, we performed the procedure with arthroscopic assistance. Arthroscopically assisted surgery allows minimal access to the coracoid process and facilitates reduction and placement of the button avoiding loss in reduction. In addition, we added the excision of the distal clavicle to facilitate the reduction, knowing that it should not affect our construction. The application of the technique we performed was completed with a double acromioclavicular repair, with allograft and coracoclavicular plasty with button sliding system, trying to provide extra support to counteract the internal ligamentous hyperlaxity.

In the other cases previously published by Barchick et al. [10] and Sahara et al. [11], less effective surgical techniques were used; therefore, they are being discarded.

The system used in our case is the Zip Tight system (Zimmer-Biomet, Warsaw, IN, USA), which allows better primary stabilization and does not generate anatomical or biomechanical alterations. The system we use has full proven efficacy in the pathology of AC dislocation, which is why we chose it to provide good primary coracoclavicular stabilization [19,20,21].

The Zip Tight system is a system that provides excellent primary fixation while allowing micro-mobility that favors proper tissue repair, avoiding excessive stiffness that interferes with proper tissue healing. It is a low-profile and knotless system that provides better tolerance in the patient since the clavicle has a thin subcutaneous coverage that makes other stabilization systems cause pain and suffering in the skin due to local pressure. 

Unlike other surgical techniques used in the other published cases [19,20,21], in our case we used tendon allograft for the biological contribution of the coracoclavicular ligamentous reconstruction and the clavicular acromion. We believe that the case we present should be treated as a chronic ligamentous injury despite the absence of pathologic findings on MRI (Figure 4).

We felt that it was necessary to treat not only the coracoclavicular but also the acromioclavicular functional insufficiency, as this corrects instability in the vertical and horizontal planes. Acromioclavicular and coracoclavicular fixation is mandatory to treat AC instability in the vertical and horizontal planes, as well as to treat associated thoracic scapular dyskinesia, which provides better scapular fixation for adequate glenohumeral mobilization. This also eliminates referred pain, allowing a symptom-free return to the patient’s daily activities and sports. 

Scapular dyskinesia is associated with both acute and chronic acromioclavicular injury. Acromioclavicular injury may be associated with isolated scapular dyskinesia or even linked to SICK scapular syndrome (scapular malposition, inferomedial border prominence, coracoid pain and malposition, and scapular motion dyskinesia), which refers to an injury resulting from overuse and muscle fatigue. 

## 5. Conclusions

The case we present is, to our knowledge, the sixth case described in the literature and the first treated by combining excision of the distal margin of the clavicle with an augmented repair. This treatment is the first that has allowed a semi-professional athlete to return to her career. Although this work is not intended to be a precise guide, the promising results obtained could help in planning the treatment of future cases.
